# Garlic peel-based carbon quantum dots as a sustainable alternative for the sensitive and green spectrofluorometric quantification of molnupiravir in pharmaceutical capsules

**DOI:** 10.1016/j.heliyon.2024.e40661

**Published:** 2024-11-26

**Authors:** Yomna A. Saber, Mahmoud Hamed, Samy Emara, Fotouh R. Mansour, Marcello Locatelli, Noha Ibrahim

**Affiliations:** a Pharmaceutical Chemistry Department, Faculty of Pharmacy, Misr International University, Km 28 Ismailia Road, Cairo, 44971, Egypt; b MIU Chemistry Society (MIU-CS), Faculty of Pharmacy, Misr International University, Km 28 Ismailia Road, Cairo, 44971, Egypt; c Pharmaceutical Analytical Chemistry Department, Faculty of Pharmacy, Tanta University, Tanta, 31111, Egypt; d Department of Analytical Chemistry, Faculty of Pharmacy, King Salman International University (KSIU), South Sinai, Egypt; e Department of Pharmacy, University “G. d’Annunzio” of Chieti-Pescara, Via dei Vestini 31, 66100, Chieti, Italy

**Keywords:** Molnupiravir, Garlic peels, Natural **c**arbon quantum dots, n-CQDs, Spectrofluorometry

## Abstract

Searching for natural alternatives to replace environmentally harmful chemical reagents in analysis is just as crucial as finding easily accessible analytical tools. To reinforce these concepts, this study proposes a simple spectrofluorometric approach using natural carbon quantum dots (n-CQDs) as fluorescence probes for sensitive and environmentally friendly measurement of molnupiravir, an antiviral drug that was initially developed for influenza and has demonstrated potential efficacy against COVID-19. n-CQDs were synthesized using garlic peels (GP), a waste material, via a microwave-assisted method. n-CQDs have a characteristic broad absorbance and narrow emission spectrum, making it easier to analyze several targets. The GP-based n-CQDs showed maximum excitation/emission at 265/347 nm with an acceptable quantum yield. After reaction with molnupiravir, the produced n-CQDs demonstrated unique features to determine the tested analyte. Different factors influencing the synthesis of n-CQDs and their interaction with the studied drug, molnupiravir, were investigated and optimized. Using GP-based n-CQDs as fluorescent probe for measuring molnupiravir byfluorescence (FL), a green analytical approach based on the probes' fluorescence quenching was developed (GP- n-CQDs -QN-FL). The method demonstrated good linearity from 0.5 to 30 μg/mL and detection/quantitation limits of 0.19/0.5 μg/mL. Validation studies confirmed accuracy (98–102 % recovery), precision (<2 % RSD), robustness and selectivity. Various assessment indexes have been utilized to assess the environmental friendliness and suitability aspects of the suggested approach in comparison to other existing techniques. Furthermore, n-CQDs were successfully employed for the precise analysis of molnupiravir in its pharmaceutical capsules. The comprehensive results proved that the method can be deemed eco-friendly and feasible more than the other techniques for its intended purpose for molnupiravir determination in pharmaceutical dosage forms with an average recovery 101.17 %.

## Graphical abstract

**Figure:**
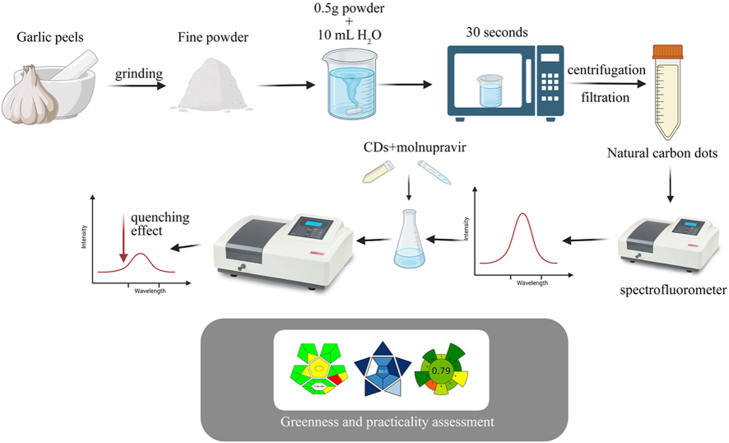

## 1 Introduction

The COVID-19 pandemic, caused by severe acute respiratory syndrome coronavirus 2 (SARS-CoV-2), a new coronavirus (CoV), has posed a significant danger to public health. More than 115 million individuals were sick by March 8th, 2021, and about 2.5 million died throughout the world [[1], [2], [3]]. In spite of the marked decline in incidence and mortality rates, COVID-19 continues to cause serious public health problems. There is still a need for effective therapeutic alternatives for controlling the epidemic. Antiviral medicines could be used as a therapy for coronavirus diseases, particularly in high-risk patient populations [4]. In this regard, one of the useful medications for therapy is molnupiravir, an antiviral agent used to treat influenza. This medication has been used for the treatment of COVID-19 disease caused by the nasopharyngeal SARS-CoV-2 infectious virus. molnupiravir has shown significant effectiveness in decreasing hospitalizations or deaths in moderate COVID-19 and might be a valuable weapon in fighting against SARS-CoV-2 [2,5,6].

Literature review showed only a few separation-based methods for molnupiravir determination either alone or in the presence of its active metabolite or other co-administered medications. These techniques included LC-MS/MS [7,8], HPLC [9], and capillary electrophoresis [10]. Unfortunately, the use of organic solvents in LC-MS/MS and HPLC can lead to environmental contamination and pose health risks to analysts if not properly disposed of. According to a recent study, an average chromatographic work may generate up to 1 L of organic waste per day, resulting in millions of liters of hazardous waste each year. Considering these potential hazards, greener analytical procedures should be developed to replace them with more environmentally friendly ones [11].

Compared to such techniques, spectrofluorometric methods are demonstrated to be simple, sensitive, time-saving, and widely available at low costs for drug assessment in various matrices [12]. As a result, developing a spectrofluorometric approach capable of sensitively determining trace amounts of molnupiravir in a simple procedure while conserving analytical time would be widely applicable [12]. Furthermore, the anticipated spectrofluorometric procedure will be entirely eco-friendly as it will employ low-energy spectrofluorimetric apparatus in lieu of various instrumental components of HPLC and LC-MS/MS as well as organic solvents [14,15]. A few analytical techniques, such as UV spectrophotometry [[13], [14], [15]] and Spectrofluorometry [16], have been developed to determine the concentration of molnupiravir either individually or in combination with other medications.

It is widely recognized that agricultural waste peels represent a major environmental challenge to society. However, these peels, being rich in lignocellulosic biomass, have opened new horizons for the development of affordable, renewable and sustainable adsorbents for water treatment purposes. Thus, garlic peels (GPs), a common agricultural waste product that is readily accessible, have the potential to serve as a cost-effective alternative to more expensive methods of wastewater treatment [17]. Within the carbon family, natural carbon-based quantum dots (n-CQDs) are a new class of nanomaterials [18]. n-CQDs have garnered significant attention from researchers due to their abundance, eco-friendly characteristics, water solubility, diverse functionalities, and biocompatibility in comparison to conventional CQDs. As a result, they are widely utilized as innovative carbon-based nanomaterials [[19], [20], [21]]. The physicochemical, and optical characteristics of n-CQDs are improved due to the presence of different functional groups on their surfaces, such as hydroxyl, carboxyl, and thiol, which makes them perfect for drug delivery, bioimaging, and sensing applications [18,22]. The n-CQDs-based photoluminescent sensors are being widely studied for their ability to detect metal ions with high sensitivity and selectivity. This is because the fluorescence quenching occurs when metal ions tightly bind to the surface of n-CQDs. Moreover, Carbon Quantum Dots have versatile applications, either independently or as composites. For instance, they can combat extended-spectrum bacteria with multidrug resistance [23,24], improve electron transport [25], facilitate hydrogen energy evolution and solar light catalysis [26], act as a bi-functional photocatalyst [[27], [28], [29]], for the development of biocompatible, antimicrobial nanomaterials for biofilm disruption [30,31].

Preparation of n-CQDs from biomass does not only help in waste management, but it also provides a cheap and sustainable source to synthesize these valuable nanosensors. Previous reports on the preparation of n-CQDs from biowaste have been found including banana stem [32], coffee [33], orange peels [34], and eggshell [35]. Similar reports were found for the preparation of n-CQDs using garlic and garlic peels for the determination of ferric ions [36], quercetin [37] and heavy metals [38]. However, these methods were time and energy consuming.

The comprehensive phytochemical profile of garlic peels underscores their potential significance as crucial components in various applications, particularly in the synthesis of n-CQDs. Notable compounds such as Quercetin, kaempferol, and rutin, alongside phenolic acids like caffeic acid, ferulic acid, and p-coumaric acid, suggesting their utility as dopants during n-CQD synthesis. Furthermore, the sulfur-containing compound S-allyl cysteine found in garlic peels adds to their therapeutic potential [39].

The preparation of n-CQDs using the microwave-assisted method offers distinct advantages over other synthesis techniques, making it highly attractive in various fields such as energy storage, bioimaging, and drug delivery [40]. One of its main benefits is the high yield of carbon dots, coupled with ease of use, cost-effectiveness, and rapid synthesis [[41], [42], [43], [44]]. This method is environmentally friendly and involves molecular-level heating, which is induced by the interaction of polar molecules with the alternating electric and magnetic fields within the solvent [45,46].

Microwave-assisted synthesis offers several practical advantages over conventional methods, including shorter reaction times and enhanced particle uniformity. While this method is energy-intensive and limited in solvent choices, it efficiently produces size-controllable nanoparticles with high yields. The rapid heating enables faster chemical bond cleavage, producing small particle sizes with narrow size distribution [47].

In the current study, an ultrafast, cost-effective and eco-friendly method of NCQDs preparation was proposed and applied as fluorescence probes for the spectrofluorometric determination of molnupiravir in dosage forms. The method was based on the quantitative fluorescence quenching of n-CQDs after the addition of molnupiravir. Compared with previous work on garlic, this method is faster, easier, simpler and more economic. The presented analytical method in this work is more suitable that the currently used HPLC methods for routine analysis of molnupiravir.

## 2 Methodology

### 2.1 Materials

Molnupiravir (99.80 %) was obtained from Eva Pharma Company (Cairo, Egypt). Sodium hydroxide and hydrochloric acid were purchased from Piochem company (6th of October, Egypt). Tryptophan (99 %) was also utilized, obtained from Alfa Chemical Group (6 industrial zone 6 of October). Molnupiravir Eva Pharma® hard capsules bearing batch number 2201179, manufactured by Eva Pharma and labeled to contain 200 mg of molnupiravir per capsule were purchased from the local market.

### 2.2 Apparatus

An RF-5301 spectrofluorometer Shimadzu (Kyoto, Japan), connected to a PC, and operated with fluorescence spectroscopy software version 2.04. The scanning parameters include an excitation and emission bandwidth of 5 nm, a scanning speed set to super, at high sensitivity. The maximum excitation wavelength (λ_ex_) was 265 nm and maximum emission wavelength (λ_em_) was 347 nm. UV absorption spectra were obtained using a shimadzu UV-1800 double beam UV–Vis spectrophotometer (Kyoto, Japan). An electron microscope, JEOL-JEM-2100(Tokyo, Japan) functioning at 80 to 200kv operating voltage was used for n-CQDs visualization and particle size measurement, and the high-resolution transmission electron microscope was performed using (HRTEM, JEOL JEM-2100 at an accelerating voltage of 200 kV). The surface charge of the n-CQDs was analyzed using zeta potential (Malvern Nano ZS, UK). A Fourier transform-infrared spectrophotometer (Bruker Optik GmbH Tensor 27, Germany) was utilized to identify the surface functional groups of the n-CQDs. A Tornado domestic microwave oven (900 W) (Banha, Egypt), Elma ultrasonic (d-78224 singen/htw) and a refrigerated centrifuge Sigma 3-16 KL (Osterode am Harz, Germany) were used during the n-CQDs synthesis. Digital analytical balance (Radwag AS 220/C/2) (Michigan, USA) was applied through the whole work. A Jenway 3510 standard digital pH Meter (Cole-Parmer, Illinois, USA) with double junction glass electrode calibrated with standard buffers was used for adjusting the pH of the solutions. X-ray diffraction data (XRD) results was obtained from the Bruker D8 Discover Diffractometer. JEOL JSM-6510 was used for Elemental mapping, and SEM imaging. Additionally, XPS analysis was conducted using a SPECS Surface Nano Analysis system (Version 4.89.2-r104748, Germany, 2022) at NRC, Egypt. The analysis was performed with high-energy resolution using specimen holders and an Al Kα X-ray source (400 W, 15 kV) with a beam size of 500 μm. A smart background correction was applied, and peak fitting was carried out using a Lorentzian-Gaussian mixed ratio of 30 %.

### 2.3 Preparation of n-CQDs sensor solution

**Fig. 1:**
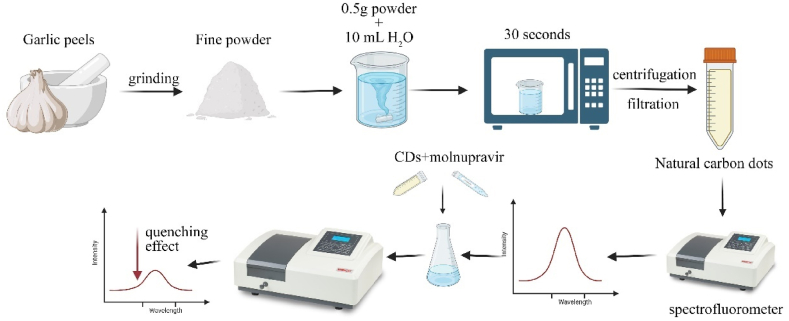
Consecutive preparation steps of natural carbon dots.

GPs utilized in this research were sourced from the vegetable market in Tanta, Egypt. Following a rinse with distilled water, the samples were air and sun-dried and subsequently crushed. The synthesis of n-CQDs was conducted using a microwave-assisted method Fig. 1. Initially, 0.5 g of crushed garlic peels powder were suspended in 10 mL of distilled water and thoroughly mixed for 2 min. The suspension was then heated in a domestic microwave at 900 W for 30 s until a pale-yellow solution was obtained. Subsequently, the solution was centrifuged at 7000 rpm for 15 min and then filtered using a syringe filter with a pore size of 0.45 μm. To ensure the removal of any remaining unfiltered particles, the n-CQDs solution underwent another round of centrifugation at 7000 rpm for 15 min. The resulting supernatant was collected in a dry, clean falcon tube and stored in a refrigerator at 4 °C for future use. The n-CQDs ' quantum yield (QY) was determined to be 3.2 %.

### 2.4 Quantum yield of n-CQDs

A single-point method was used for the determination of the quantum yield of the n-CQDs using the following equation.

QY_CQD_ = QY_ST_ X (F_CQD_/F_ST_)X(A _ST_/A _CQD_)X(η_CQD_/η_ST_)^2^

F represents the integrated fluorescence intensity, A represents the absorbance, and η is the solvent refractive index. Tryptophan is used as a reference standard using phosphate buffer (100 mM, pH 7) as a solvent. we selected a concentration that kept the standard absorbance lower than 0.05.

### 2.5 Molnupiravir determination using n-CQDs

General procedures for molnupiravir detection: Different aliquots (25–1500 μL) of molnupiravir stock solution (100 μg/mL) and 800 μL of n-CQDs solution were transferred to 5 mL calibrated flask, then the volume was completed to the final volume with the distilled water to obtain final molnupiravir concentrations in the range of 0.5–30 μg/mL. The solution was mixed and fluorescence intensities (*F*) were measured at 347 nm after excitation at 265 nm. A calibration curve was constructed by plotting *F*^*0*^*-F* versus the corresponding molnupiravir concentration, where *F*^*0*^ and *F* are the fluorescence intensity of n-CQDs before and after addition of molnupiravir, respectively.

For dosage form assay, ten molnupiravir capsules were emptied and the powder was weighed then mixed well. An average weight of one capsule was transferred into 100 mL volumetric flask, 40 mL of distilled water was added and sonicated for 15 min with shaking each 5 min till full dissolving, then left till reaching room temperature and the volume was then completed to 100 mL with distilled water to obtain a final concentration of 2 mg/mL. A second diluted stock solution of 200 μg/mL is prepared by appropriate dilution of the previous solution with distilled water. Different volumes of the working stock solution were transferred to 5 mL volumetric solution to prepare samples for analysis.

## 3 Results and discussion

### 3.1 Preparation and characterization of n-CQDs

An innovative bio-compatible and simple microwave-assisted technique for manufacturing n-CQDs from GPs is demonstrated, providing a more effective and the least hazardous alternative to existing methods. The resulting n-CQDs boast impressive optical characteristics such as blue fluorescence, and remarkable fluorescence stability. When combined with molnupiravir, the synthesized n-CQDs create a reliable nano-probe for measuring the analyte in pure and pharmaceutical capsule forms with high sensitivity. This study not only presents a novel approach to molnupiravir sensing and measurement in pharmaceutical samples but also introduces a sustainable method for managing biomass waste. In comparison to alternative bottom-up strategies, the use of microwave irradiation can lead to a decrease in reaction duration to minutes instead of the hours (2–12 h) typically associated with the hydrothermal method. The duration of the reaction was analyzed, revealing that the optimal reaction period was determined to be 30 s. A shorter period of heating proved insufficient for achieving carbonization, while a longer duration did not yield improved results. Subsequent to their preparation, the n-CQDs underwent purification through centrifugation and filtration to eliminate any particles or residues. Additionally, multiple cycles of n-CQDs preparation were executed, resulting in consistently reproducible fluorescence intensity readings.

**Fig. 2:**
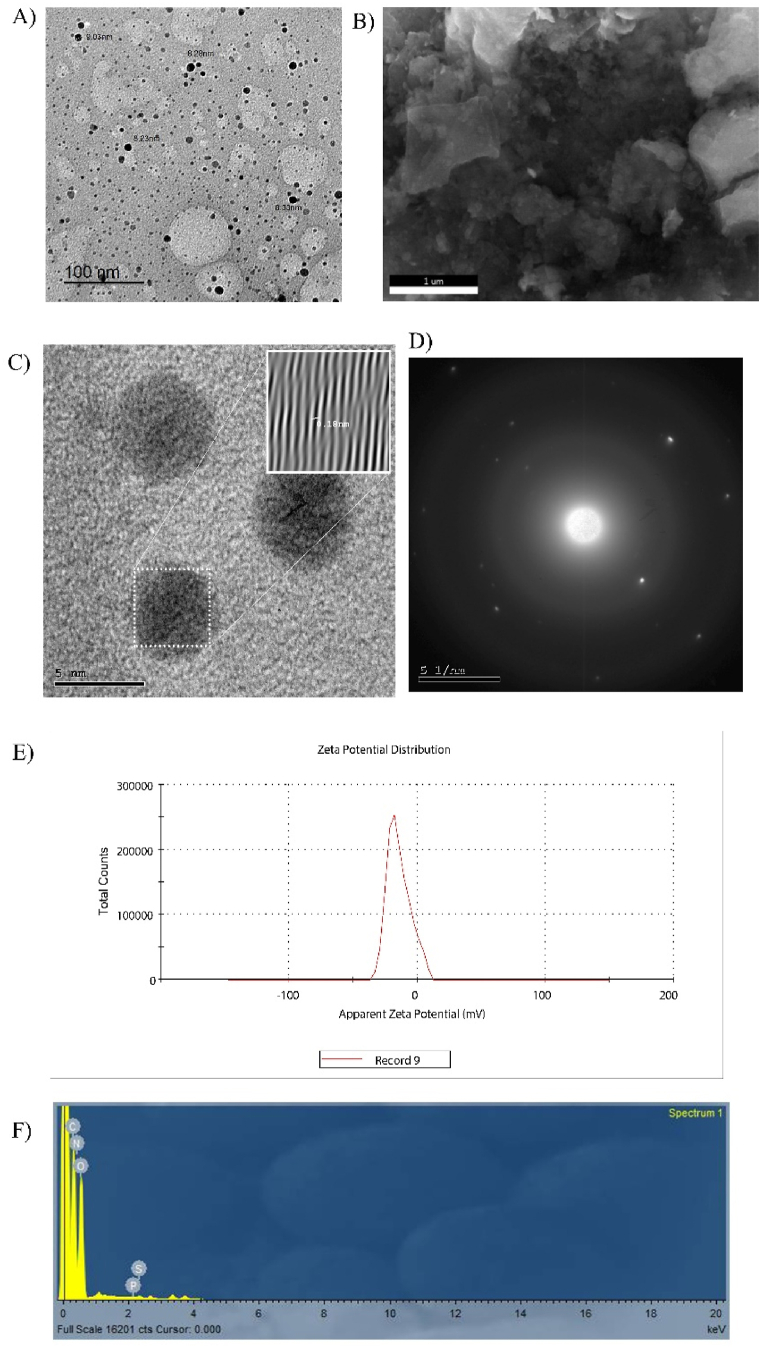
A) The TEM image of the synthesized n-CQDs under a magnification power of 100 nm, B) Scanning electron microscopy images (SEM), C, D) shows a high-resolution TEM image of CQD, illustrating the crystallinity of the CQDs and shows spacing of 0.18 nm E) Zeta potential distribution of the n-CQDs, F) EDX elemental analysis of the n-CQDs.

**Fig. 3:**
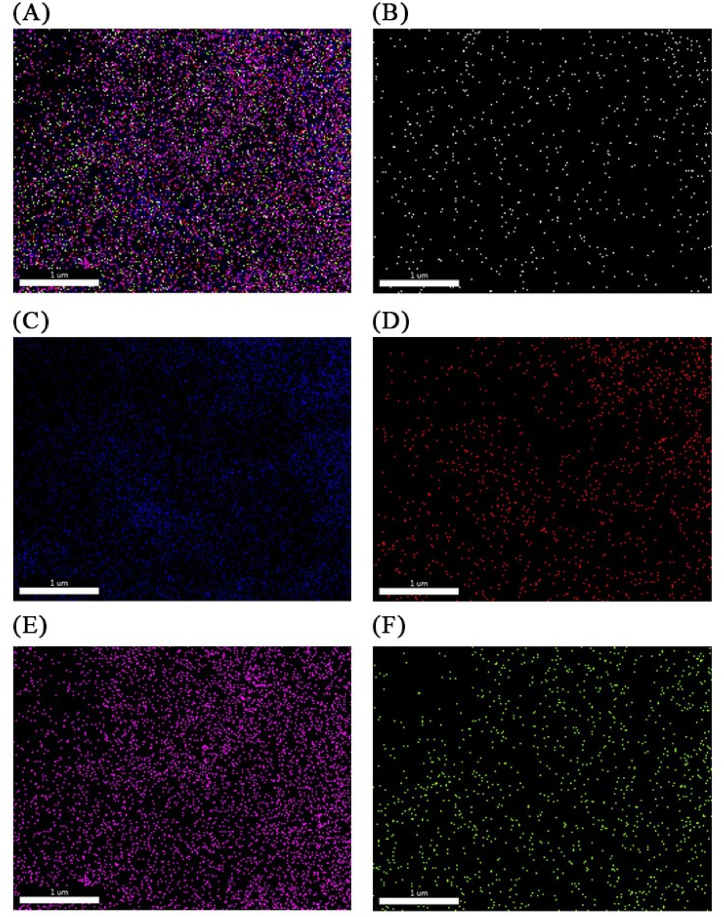
Elemental mapping illustrates the distribution of (A) carbon, oxygen, nitrogen, sulfur, and phosphorus in an overlay, followed by individual element maps: (B) carbon, (C) oxygen, (D) nitrogen, (E) sulfur, and (F) phosphorus in n-CQDs.

**Fig. 4:**
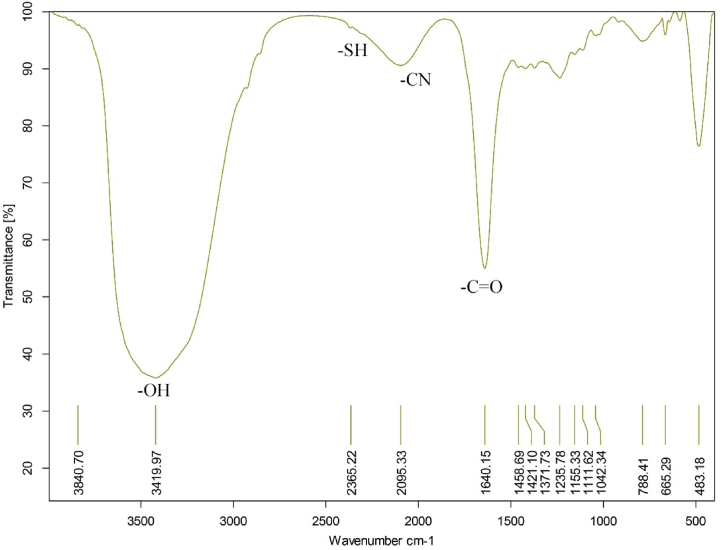
FTIR spectrum of the synthesized n-CQDs.

The fluorescence spectrum of n-CQDs solution was recorded to characterize the prepared n-CQDs. As shown in Fig. S1, n-CQDs have absorption peaks at 265 nm due to π-π∗. The maximum emission was observed at an excitation wavelength of 347 nm. Fig. 2(A–C and D) shows the TEM and HR-TEM image of the synthesized n-CQDs. It shows that the n-CQDs are homogenously distributed, mono-dispersed, nearly spherical shaped with an average diameter of about 2–4 nm and mainly distributed in the range of 1–9 nm with crystalline structure and interlayer spaces of 0.18 nm Fig. S2. These particles are mono-dispersed due to the presence of negative charges on the surface. The SEM image of the n-CQDs exhibits a porous structure with an irregular surface morphology, as illustrated in Fig. 2(B). The zeta potential value of n-CQDs was determined to be −14.5 mV Fig. 2(E), which indicates the possible presence of negatively charged carboxyl (–COOH), hydroxyl (–OH), and (-CN) functional groups on their surface. The surface morphology with elemental composition Fig. 2(F) was reassessed using EDX elemental mapping. The EDX pattern of n-CQDs asserts the existence of carbon(C), oxygen (O) and nitrogen (N) elements with the following weights 29.85, 38.54, 31.54 %, respectively and minor amounts of sulfur (S) and phosphorus (P). The absence of any other elements in the EDX spectrum suggests that the synthesis processes did not bring any impurities. The elemental mapping Fig. 3 shows that there is uniform distribution of C, O, N, S, and P throughout. The FT-IR spectrum of the GP-based n-CQDs was conducted to verify the functional groups. Fig. 4 demonstrates that the primary characteristic bands of the n-CQDs were observed at 3419.97 cm^−1^ for -OH stretching and 1640.15 cm^−1^ for -C=O stretching. Furthermore, additional bands were detected at 2095.33 cm^−1^ (-CN) and 2365.22 cm^−1^ (-SH).

To discuss the XRD Fig. S3 spectrum for natural carbon quantum dots (n-CQDs), we can examine their structural features and crystallinity. The n-CQDs typically exhibit either a graphitic crystalline structure (sp^2^) or an amorphous form (mixed sp^2^/sp^3^) based on the presence of sp^2^ carbon in their core. The crystallinity of CQDs, particularly in n-CQDs, is influenced by the extent of graphitic carbon domains and amorphous regions. In Fig. S3 the XRD spectrum of n-CQDs shows a broad peak around the 2θ range of 18°–24°, which aligns with the diffraction plane of graphitic carbon. This broad characteristic peak indicates that n-CQDs possess poor crystallinity, which is common due to the introduction of oxygen-containing groups during synthesis. The broadening of the XRD peak also suggests that the size of the graphitic domains within the n-CQDs is small, consistent with the nanometer-scale structure of CQDs.

**Fig. 5:**
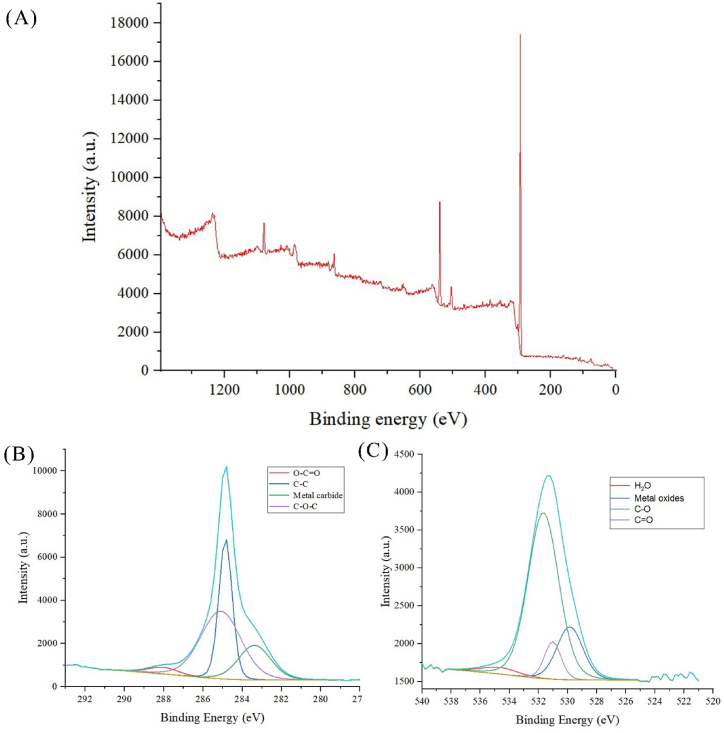
(A) XPS survey spectrum of n-CQD, high resolution scans of (B) C1s, and (C) O1s.

X-ray Photoelectron Spectroscopy (XPS) offers valuable insights into the presence of surface functional groups in n-CQDs. In Fig. 5(A), the XPS survey spectrum of n-CQDs reveals distinct peaks at 284.7 eV and 531.7 eV, corresponding to the binding energies of C1s and O1s, respectively. Upon closer examination in the high-resolution scan of the C1s region Fig. 5(B), the spectrum illustrates the presence of carbons in four distinct chemical environments. Specifically, peaks are observed at 288.5 eV for O-C=O, at 285 eV for C-O-C, at 284.8 eV for C-C bonds, and at 283 eV for metal carbides. Furthermore, in Fig. 5(C), the O1s core-level peaks are identified at 529.5, 531.5, 532.5, and 535 eV, indicative of various chemical bonds. These peaks are attributed to metal oxides, Organic C=O bonds, Organic C-O bonds, and H_2_O bonds, respectively.

### 3.2 Studying the quenching mechanism

Fig. S2 shows the UV spectrum for n-CQDs, molnupiravir, and n-CQDs with molnupiravir, demonstrating that the UV absorbance of n-CQDs increases upon adding molnupiravir. This increase in absorbance indicates either static or dynamic quenching of fluorescence, likely due to spectral overlap effects. To further investigate, the temperature effect was analyzed, which suggested static quenching. The Stern-Volmer plot exhibited good linearity at different molnupiravir concentrations Fig. S5 at two different temperatures 30 °C and 40 °C, and it was noticed that increasing the temperature leads to decreases the Stern-Volmer quenching constant (Ksv) which can be explained by dissociating of the formed complex. The Stern-Volmer equation used is:

$$\frac{F^{0}}{F}=1+K_{sv}\left[Q\right]$$

Here, *F*^*0*^ and *F* represent the fluorescence emission intensities of n-CQDs in the absence and presence of molnupiravir, respectively. *Ksv* is the Stern-Volmer quenching constant, and [Q] is the concentration of molnupiravir.

### 3.3 Optimization of n-CQDs/molnupiravir measurement

In order to check the better performance of the developed n-CQDs fluorescence probes in molnupiravir measurement, several parameters should be optimized, including pH, incubation time, and n-CQDs volume. The initial investigation was conducted using a solution containing 10 μg/mL of molnupiravir and 800 μL of n-CQDs using distilled water as a solvent. The optimization study focused on analyzing all the factors influencing the interaction between the n-CQDs and molnupiravir, with a systematic examination of each factor individually while keeping the other factors constant.

#### 3.3.1 Effect of pH

**Fig. 6:**
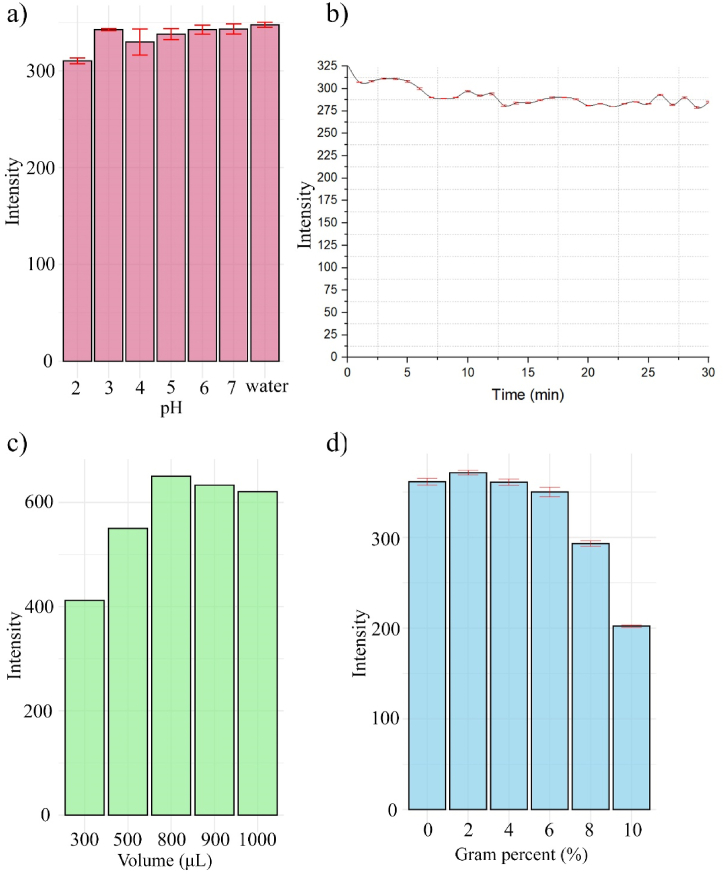
The effect of diluent pH (A), mixing time (B), n-CQDs volume (C) and ionic strength (D) on the fluorescence intensity of the synthesized n-CQDs (±SD, n = 3).

The pH has an important role as it does not only affect the fluorescence intensities but also the interactions between n-CQDs and the tested analyte. Thus, pH optimization was carried out within the range of 2.0–7.0 by utilizing hydrochloric acid to explore this specified range. Sodium hydroxide was utilized for pH adjustment to the desired values. It was observed that a slight decrease in fluorescence intensity occurred at the lowest pH value (pH 2). This phenomenon can be attributed to the protonation of the hydroxyl groups on the surface of the n-CQDs, leading to the formation of hydrogen bonds and subsequent aggregation of the n-CQDs, resulting in a reduction in fluorescence intensity Fig. 6 (A). Additionally, optimal results and excellent performance of the n-CQDs were achieved within the pH range of 5–7, further confirming the reliability of the developed method. The choice of distilled water as the solvent was based on the excellent fluorescence stability of n-CQDs, and it is frequently regarded as an optimal environmentally friendly solvent.

#### 3.3.2 Effect of incubation time

The impact of incubation time on the fluorescence intensity of the quenching reaction of molnupiravir on the n-CQDs was investigated by observing the fluorescence intensity of n-CQDs during the specified time period after introducing the standard analyte. This assessment spanned 30 min, with readings taken at 1-min intervals. It was noticed that, n-CQDs exhibited non instantaneous fluorescence quenching at 347 nm following excitation at 265 nm when interacting with molnupiravir Fig. 6 (B) and required 7 min to attain a stable fluorescence intensity. Furthermore, fluorescence emission remained unchanged even when the reaction time increased up to 9 min. An incubation time of 8 min was selected to reach completion and stabilize the fluorescence intensity to achieve accurate results.

#### 3.3.3 n-CQDs volume

The impact of varying n-CQDs volume, ranging from 300 to 1000 μL, was examined to determine the optimal F0 value. The findings revealed a steady F0 across the range of 800–900 μL of n-CQDs volume. Consequently, the optimal volume of 800 μL was chosen, as depicted in Fig. 6 (C).

#### 3.3.4 Ionic strength

Various amounts of sodium chloride solution were added to explore how ionic strength affects the performance of n-CQDs, as presented in Fig. 6 (D). The results revealed that small additions (2 % and 4 %) sodium chloride had a negligible impact on fluorescence intensity. However, with larger amounts (over 6 %), the fluorescence intensity rose, suggesting the presence of ionized groups on the n-CQDs’ surfaces. Accordingly, no salt addition was recommended in the following procedure.

### 3.4 Method validation

The suggested approach underwent validation in accordance with ICH guidelines [48], assessing aspects such as linearity, range, detection and quantification limits, accuracy, precision, and robustness.

#### 3.4.1 Linearity and range

**Fig. 7:**
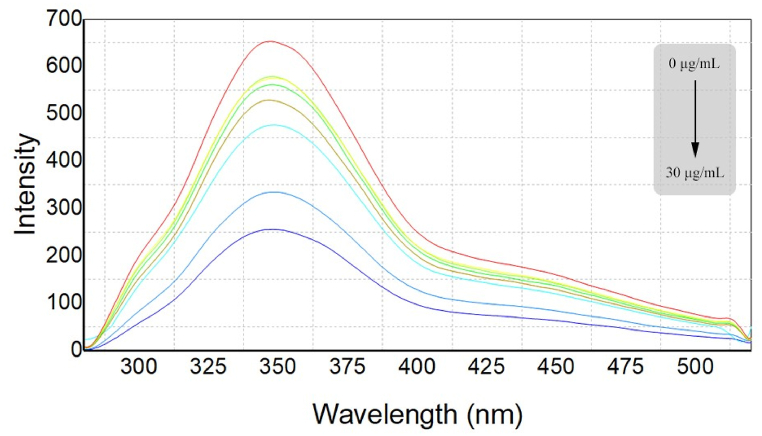
The fluorescence emission spectra of n-CQDs under varying concentrations of molnupiravir (0.5–30 μg/mL).

The fluorescence spectra of n-CQDs were measured both prior to and following exposure to various concentrations of molnupiravir ranging from 0.5 to 30 μg/mL Fig. 7. The n-CQDs solution was mixed with molnupiravir standard solutions under optimal experimental conditions. As depicted in Fig. 7, molnupiravir has the ability to quench the fluorescence intensity of n-CQDs, whereas, the fluorescence spectrum of the n-CQDs solution remained largely unchanged in terms of bandwidth and maximum emission wavelength Fig. 7. Additionally, a linear correlation was found between the concentration of molnupiravir and the n-CQDs' quenching fluorescence intensity Table 1. These results highlight the potential utility of a n-CQDs-based fluorescence probe for accurately measuring molnupiravir.

**Table 1:** The regression parameters of the GP- n-CQDs -QN-FL method.

Parameters	
**Linear range (μg/mL)**	0.5–30
**Slope ± Sb**	11.5 ± 0.4
**Intercept ± Sa**	63.07 ± 5.9
**Coefficient of determination (r2)**	0.991
**LOD (μg/mL)**	0.19
**LOQ (μg/mL)**	0.5

**N.B.:** S_a_: standard error of intercept, S_b_: standard error of slope, LOD and LOQ were calculated.

#### 3.4.2 Limit of detection and limit of quantification

The limits of detection (LOD) and quantification (LOQ) were determined using the standard equations: LOD = 3.3 × (σ/S) and LOQ = 10 × (σ/S), where σ is the standard deviation of blank samples and S is the slope of the calibration curve Fig. S6. The calculated LOD (0.19 μg/mL) and LOQ (0.5 μg/mL) indicate the method's high sensitivity, allowing for the reliable detection and quantification of low concentrations of molnupiravir. The method's linear range of 0.5–30 μg/mL, along with a high coefficient of determination (r^2^ = 0.991), demonstrates strong linearity and accuracy across the tested concentration range. These parameters ensure the method is suitable for sensitive and reliable molnupiravir analysis in various applications.

#### 3.4.3 Trueness and precision

To assess the accuracy of the proposed method, we determined three different concentrations of molnupiravir within the linearity range in triplicate. Trueness was evaluated by calculating the % recovery, which fell within the accepted range of 98–102 % according to Table 2. This indicates that the results are close to the true value.

**Table 2:** Evaluation of the Trueness and Precision of the GP- n-CQDs -QN-FL method.

Concentration taken μg/mL	Trueness and intraday precision				Inter-day precision		
	Mean concentration found^a^ μg/mL	SD	RSD %	Recovery %	Mean concentration found∗ μg/mL	SD	RSD %
**2**	2.02	0.03	1.3	100.90	2.01	0.01	0.599
**8**	7.90	0.06	0.82	98.78	99.03	0.05	0.643
**16**	16.27	0.03	0.17	101.70	100.97	0.10	0.632
Mean %recovery ±SD				100.46 ± 1.23			

N.B.

a n = 3.

The precision of the GP- n-CQDs -QN-FL method was measured by evaluating both intraday and inter-day precision. For intraday precision, three replicates of three different molnupiravir concentrations within the linearity range were determined on the same day. Similar procedures were repeated on three consecutive days to estimate inter-day precision. All results, summarized in Table 2, showed a %RSD less than 2 %, indicating a precise method.

#### 3.4.4 Robustness

To evaluate the GP- n-CQDs -QN-FL method's robustness, we investigated the influence of slight, intentional variations in fluorescence response parameters. These parameters included pH (±0.2), incubation time (varied by 1.0-min intervals), emission wavelength (347 ± 2.0 nm), and excitation wavelength (265 ± 2.0 nm). As shown in Table 3, these minor changes did not affect the method's performance, indicating a robust method (see Table 4).

**Table 3:** Evaluation of the robustness of the GP- n-CQDs -QN-FL method.

Parameter	Conditions	Conc. In sample μg/mL	Mean Conc. found^a^	%Recovery	Mean %Recovery	%RSD
**pH**	5.80	10.00	10.03	100.32	99.36	0.90
	6.00	10.00	10.10	99.60		
	6.20	10.00	9.82	98.16		
**Emission wavelength(nm)**	345.00	10.00	10.12	101.24	100.61	0.50
	347.00	10.00	10.06	100.60		
	349.00	10.00	10.00	100.00		
**Excitation wavelength (nm)**	263.00	10.00	10.17	101.67	100.76	0.68
	265.00	10.00	10.06	100.60		
	267.00	10.00	10.00	100.01		
**Incubation time (min)**	7.00	10.00	10.00	100.00	100.10	0.19
	8.00	10.00	10.03	100.36		
	9.00	10.00	9.99	99.94		

N.B.

a n = 3.

**Table 4:** Recovery study using standard addition method.

	Recovery level (%)	Initial concentration (μg/mL)	Added concentration (μg/mL)	% recovery	SD	%RSD
Molnupiravir	100 %	10	10	101.17	2.5	0.7

N.B.: ∗n = 3.

#### 3.4.5 Selectivity

**Fig. 8:**
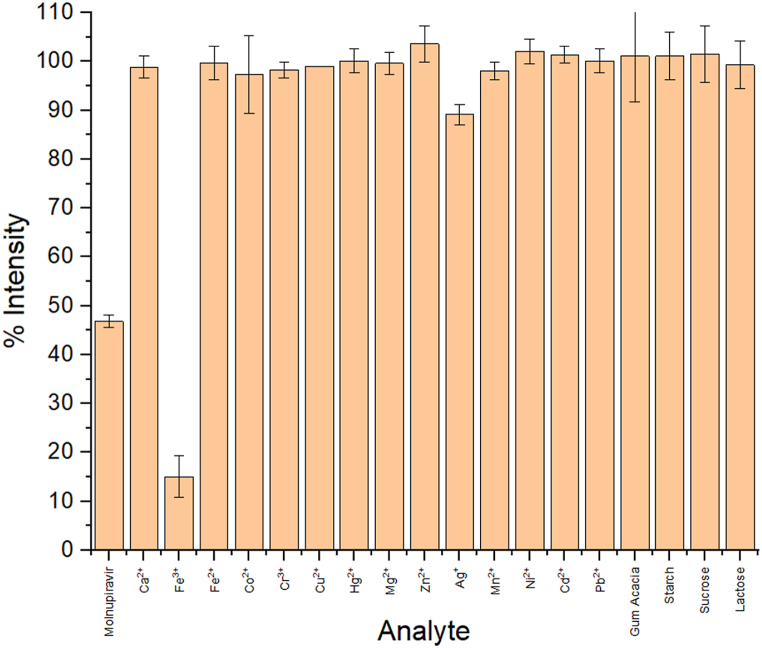
Evaluation of the selectivity of n-CQDs to a wide range of metal ions and commonly used excipients in dosage form formulation (±SD, n = 3).

Metal complexation is a prevalent characteristic observed in the majority of CQDs. Consequently, the impact of various metal ions, including Ca^2+^, Fe^3+^, Fe^2+^, Co^2+^, Cr^3+^, Cu^2+^, Hg^2+^, Mg^2+^, Zn^2+^, Ag^+^, Mn^2+^, Ni^2+^, Pb^2+^, and Cd^2+^, on the fluorescence intensities of n-CQDs was assessed. It was observed that Fe^3+^ exhibited the most pronounced quenching effect on the fluorescence intensities of the n-CQDs, whereas Ag^+^ had a minimal effect, as depicted in Fig. 8. Furthermore, commonly utilized excipients in dosage form formulations, such as acacia gum, starch, sucrose, fructose, and lactose, were investigated for potential interference and demonstrated no significant impact on the fluorescence intensities of the n-CQDs.

### 3.5 Application of the GP- n-CQDs -QN-FL method for dosage form analysis

To determine the analyte concentration in the dosage form, molnupiravir capsules were processed to prepare a stock solution (2 mg/mL), which was then diluted to obtain a working solution (200 μg/mL). A final concentration of 10 μg/mL molnupiravir was prepared by diluting the working solution and adding n-CQDs. The method's applicability was confirmed using the standard addition method, achieving an average recovery of 101.17 % and an RSD of 0.7 %.

### 3.6 Green assessment of the proposed method

**Fig. 9:**
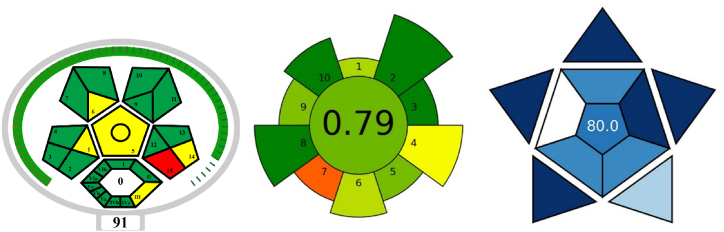
Greenness and practicality assessments of the GP- n-CQDs-QN-FL method using different matrices such as ComplexMoGAPI, AGREEprep, and BAGI.

The environmental friendliness of the GP- n-CQDs -QN-FL method was evaluated through the use of accepted tools for this purpose [28], specifically ComplexMoGAPI [49,50], and AGREEprep [51], while practicality was assessed through the BAGI index [52] to determine its environmental sustainability, compatibility and effectiveness. The pictograms shown in Fig. 9 highlight the greenness of the method. The ComplexMoGAPI shows a green method except for the 15th parameter (Waste treatment), as the whole prepared sample is considered waste after analysis using Spectrofluorometry, while the BAGI pictogram proves the practicality of the method. The only concern regarding the method is that according to the 2nd parameter (single of multi element analysis) the method provides a single component analysis. While the only major concern regarding the AGREEprep is that the method requires multiple manual steps before getting the sample ready for analysis. Despite these concerns, the overall results of the greenness assessment and practicality prove that the method is considered green and reliable for its intended use.

### 3.7 Comparison with other reported methods

Here we provide a comparison between our proposed method and previous published method for the determination of molnupiravir Table 5. It is obvious that the GP- n-CQDs-QN-FL method provide comparable results in terms of LOD and linearity range. But what makes our method relatively better, is that the method is simple using spectrofluorimetry, cost effective, depending on natural waste material for the preparation of the n-CQDs and water as a solvent, and require no extensive sample preparation.

**Table 5:** A comparison between the GP-n-CQDs-QN-FL method and previously published methods.

Reported method	Analyte	Molnupiravir Linearity (μg/mL)	Molnupiravir LOD (μg/mL)	MoGAPI	AGREE	Analytical Eco-Scale	Matrix	Ref.
LC-MS/MS	Molnupiravir and its metabolite	0.025–5	NR			71	human plasma and saliva	[8]
Spectrophotometric	Molnupiravir	2.5–20.0	0.37			81	Bulk Powder and Pharmaceutical Formulation	[53]
Micellar HPLC	Molnupiravir and Favipiravir	0.5–50.0	0.02			73	Favipiravir tablets and molnupiravir capsules	[16]
High-performance thin layer chromatography	Favipiravir, molnupiravir, and ritonavir	3.75–100.00	1.21			66	laboratory prepared capsules	[54]
Spectrofluorometry	Molnupiravir	2.50–70.00	0.80			93	Pharmaceutical dosage form	[35]
spectrophotometric	Molnupiravir	1.00–12.0	NR			79	Pharmaceutical dosage form	[13]
Spectrofluorometry	Molnupiravir	0.5–30	0.19			93	Pharmaceutical dosage form	This work

## 4 Conclusion

A new, fast, innovative, and green spectrofluorimetric technique was established to quantify molnupiravir. This method relies on the ability of the fluorescence intensity of n-CQDs to be quenched by the analyte being tested in distilled water. The study also delved into the different factors that affect fluorescence quenching. The linear relationship between molnupiravir concentration and the fluorescence quench effect of the n-CQDs, which served as a platform for quantifying the tested analyte was achieved within the range of 0.5–30 μg/mL. The synthesized n-CQDs exhibited unique features, allowing for sensitive quantification of molnupiravir both its pure form and pharmaceutical capsules. The proposed GP- n-CQDs -QN-FL method offers several advantages, including simplicity, sensitivity, cost-effectiveness, and reduced environmental impact compared to traditional techniques involving organic solvents. The use of n-CQDs as a fluorescent probe holds promise for the determination of other pharmaceuticals and biologically active compounds. This research contributes to the development of greener analytical procedures for drug assessment, facilitating the fight against the COVID-19 pandemic and potentially benefiting future antiviral drug research. Also, the method's practicality results demonstrate that, it is feasible for its intended use. This innovative GP-n-CQDs-QN-FL approach demonstrates comparable performance to other established methods. While eliminating the need for harmful organic solvents, the proposed method also offers a simple and cost-effective alternative for molnupiravir determination in both pure and pharmaceutical forms.

## CRediT authorship contribution statement

**Yomna A. Saber:** Writing – original draft, Investigation, Formal analysis, Data curation. **Mahmoud Hamed:** Writing – review & editing, Writing – original draft, Visualization, Validation, Supervision, Methodology, Formal analysis, Conceptualization. **Samy Emara:** Writing – review & editing, Software, Project administration, Conceptualization. **Fotouh R. Mansour:** Writing – review & editing, Writing – original draft, Software, Project administration, Conceptualization. **Marcello Locatelli:** Writing – review & editing, Writing – original draft, Supervision, Conceptualization. **Noha Ibrahim:** Writing – review & editing, Supervision, Project administration, Methodology, Conceptualization.

## Data availability statement

Data will be made available on request.

## Funding

Marcello Locatelli acknowledges financial support funded by the 10.13039/501100000780European Union – NextGenerationEU, under the National Recovery and Resilience Plan (NRRP), Mission 4 Component 2 - M4C2, Investment 1.5 – Call for tender No. 3277 of December 30, 2021, Italian Ministry of University, Award Number: ECS00000041, Project Title: “Innovation, digitalization and sustainability for the diffused economy in Central Italy”, Concession Degree No. 1057 of June 23, 2022 adopted by the Italian Ministry of University. CUP: D73C22000840006.

## Declaration of competing interest

The authors declare no conflict of interests.
