# Supplementary material for: Garlic peel-based carbon quantum dots as a sustainable alternative for the sensitive and green spectrofluorometric quantification of molnupiravir in pharmaceutical capsules
Source: Heliyon. 2024 Nov 26;10(23):e40661. doi: 10.1016/j.heliyon.2024.e40661 (PMC11652926; doi:10.1016/j.heliyon.2024.e40661)
Supplement: Multimedia component 1 [file mmc1.docx]

**Garlic Peel-based Carbon Quantum Dots as a Sustainable Alternative for the Sensitive and Green Spectrofluorometric Quantification of Molnupiravir in Pharmaceutical Capsules**

*Yomna A. Saber^1^, Mahmoud Hamed^1,2^, Samy Emara^1,2^, Fotouh R. Mansour^3,4*^, Marcello Locatelli^5*^, Noha Ibrahim^1,2^*

*^1^Pharmaceutical Chemistry Department, Faculty of Pharmacy, Misr International University, Km 28 Ismailia Road, Cairo 44971, Egypt*

*^2^MIU Chemistry Society (MIU-CS), Faculty of Pharmacy, Misr International University, Km 28 Ismailia Road, Cairo 44971, Egypt.*

*^3^Pharmaceutical Analytical Chemistry Department, Faculty of Pharmacy, Tanta University, Tanta, 31111, Egypt*

*^4^Department of Analytical Chemistry, Faculty of Pharmacy, King Salman International University (KSIU), South Sinai, Egypt*

*^5^ Department of Pharmacy, University “G. d’Annunzio” of Chieti-Pescara, Via dei Vestini 31, 66100 Chieti, Italy*

**Running title:** Green n-CQDs for Molnupiravir Determination

**Fotouh R Mansour, BSc (Hons), MSc, MBA, PhD
Department of Pharmaceutical Analytical Chemistry
Faculty of Pharmacy, Tanta University
Tanta 31111, El-Gharbiya, EGYPT
e-mail:**[**Fotouhrashed@pharm.tanta.edu.eg**](mailto:Fotouhrashed@pharm.tanta.edu.eg)

**Prof. Marcello Locatelli**

**Department of Pharmacy**

**University “G. d’Annunzio” of Chieti-Pescara**

**Via dei Vestini 31**

**66100 Chieti**

**Italy**

**E-mail:** [**marcello.locatelli@unich.it**](mailto:marcello.locatelli@unich.it%20)


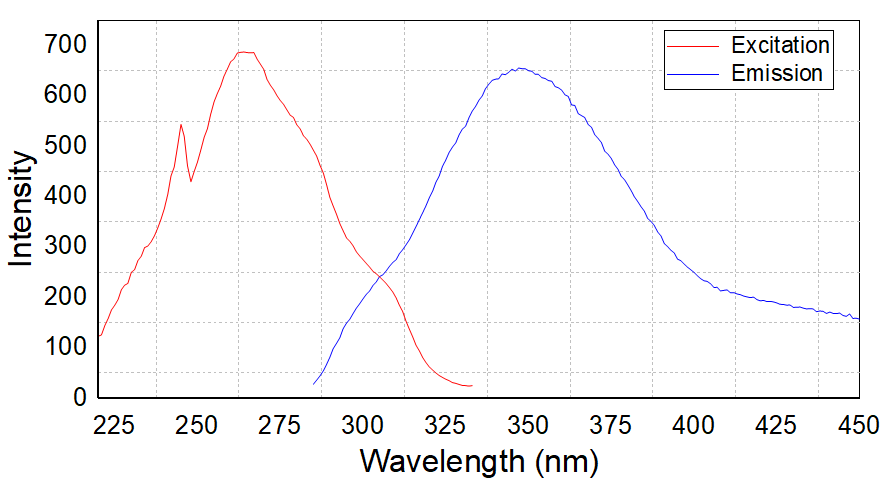


**Figure S1:** The excitation and emission wavelengths observed in CDs overlaid.


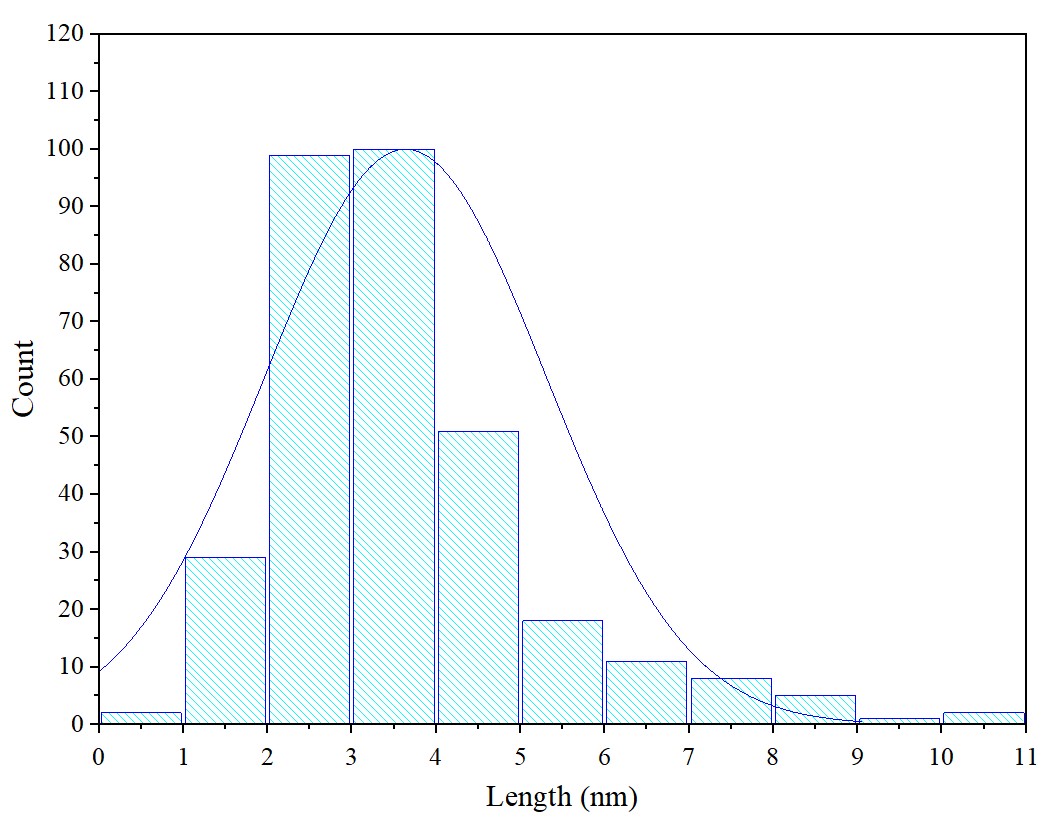


**Figure S2:**Particle size distribution histogram of n-CQDs


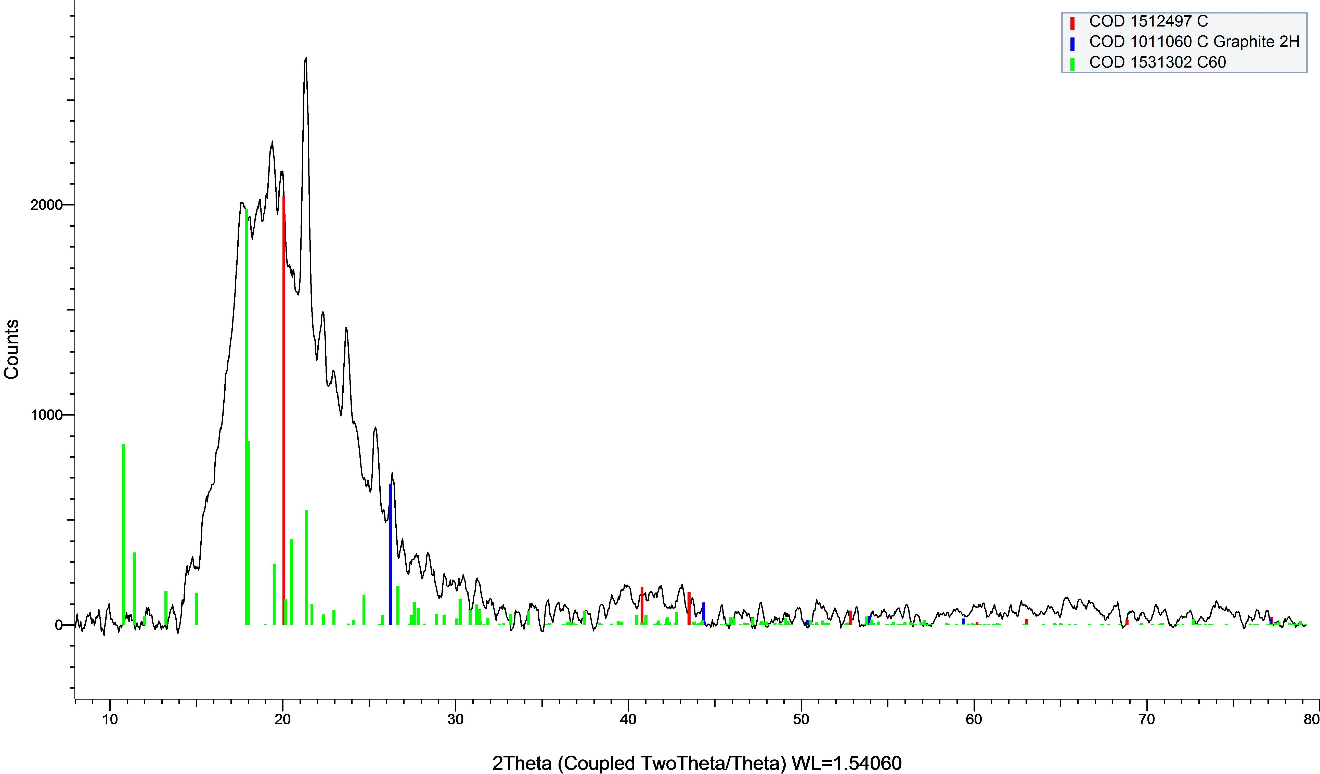


**Figure S3:**XRD of pattern of n-CQDs.

*
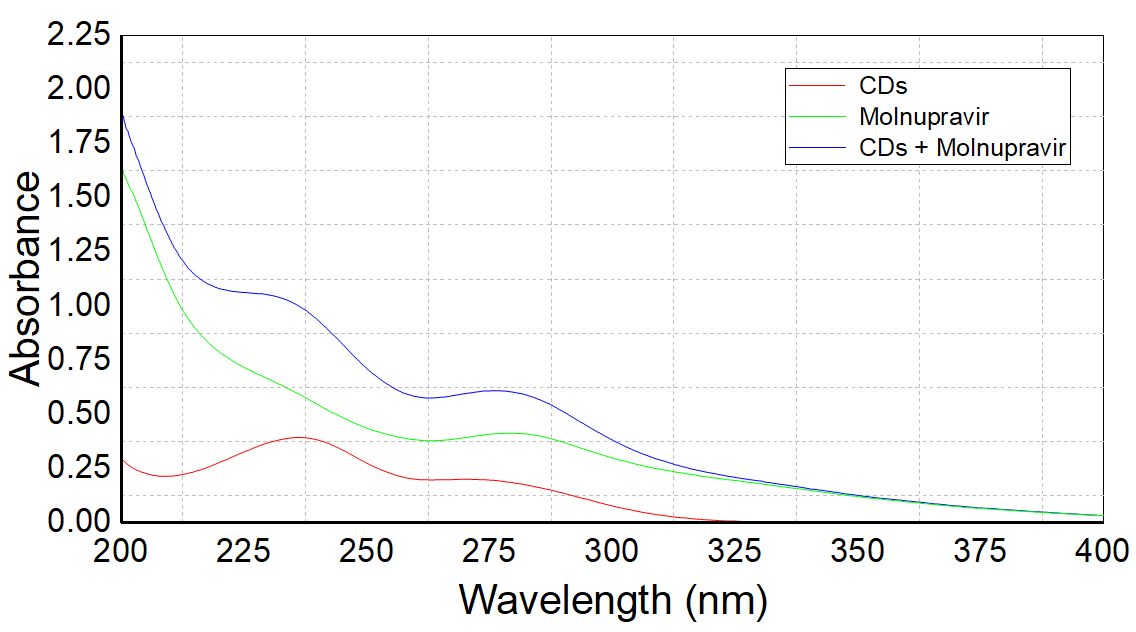
*

**Figure S4:** Uv spectrum for the synthesized CDs , molnupiravir, and the mixture of both CDs and molnupiravir.


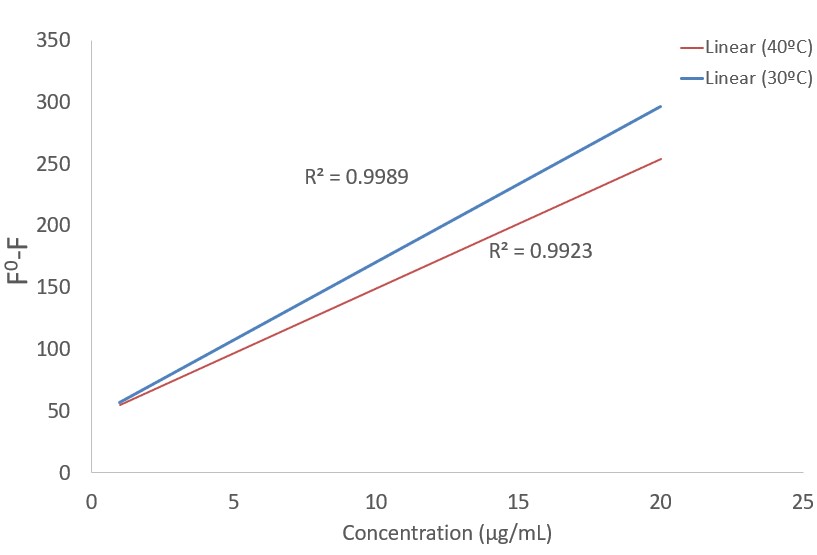


**Figure S6:** Stern–Volmer plots for n-CQDs fluorescence quenching by molnupiravir at two different temperatures (30 °C and 40 °C).


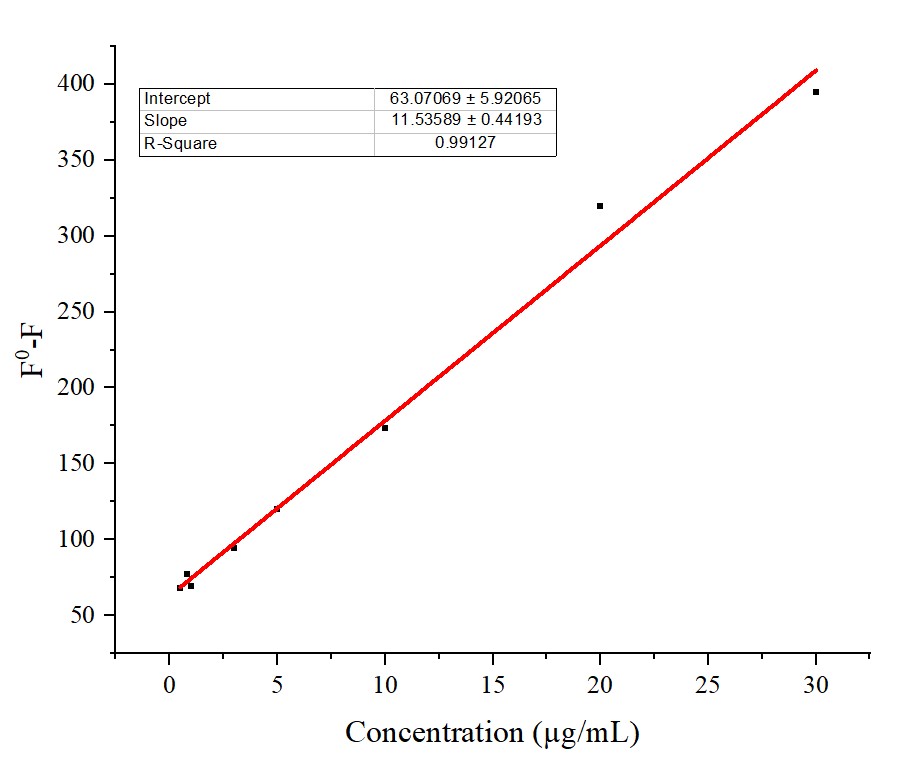


**Figure S5:** Calibration curve for the GP- n-CQDs -QN-FL method.

**
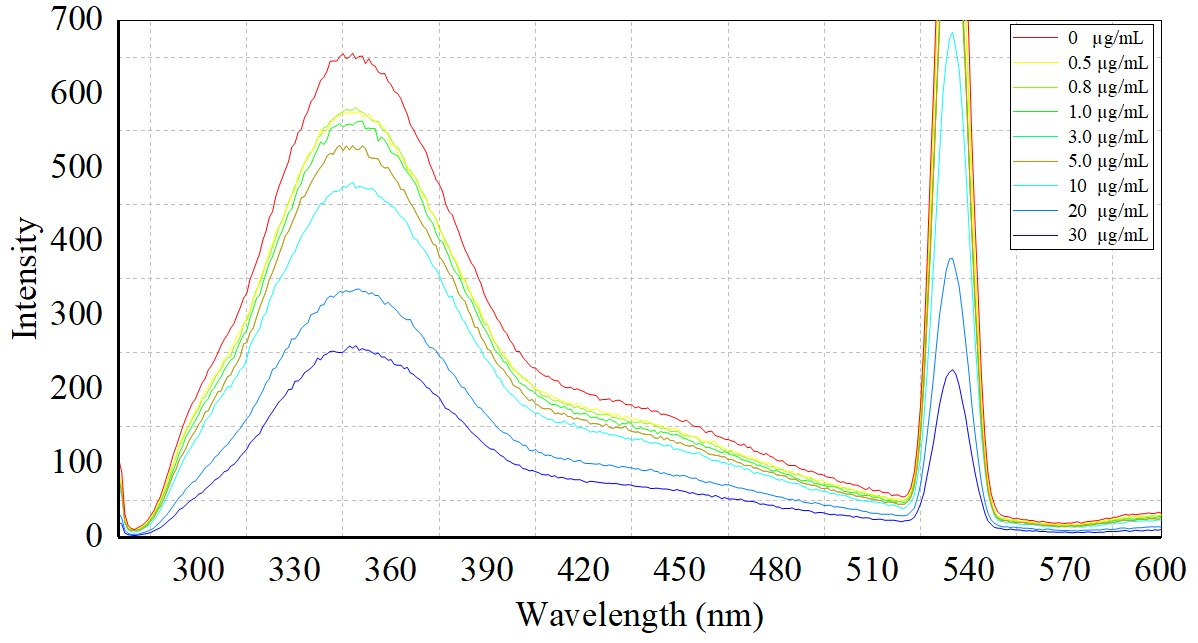
**

**Figure S6:** The full fluorescence emission spectra of n-CQDs under varying concentrations of molnupiravir (0.5–30 µg/mL).
